# Anti-inflammatory effects induced by pharmaceutical substances on inflammatory active brain astrocytes—promising treatment of neuroinflammation

**DOI:** 10.1186/s12974-018-1361-8

**Published:** 2018-11-17

**Authors:** Elisabeth Hansson, Ulrika Björklund, Eva Skiöldebrand, Lars Rönnbäck

**Affiliations:** 1 Department of Clinical Neuroscience, Institute of Neuroscience and Physiology, The Sahlgrenska Academy, University of Gothenburg, Blå Stråket 7, 3rd floor, SE 413 45 Gothenburg, Sweden; 2 Section of Pathology, Department of Biomedical Sciences and Veterinary Public Health, Swedish University of Agricultural Sciences, Uppsala, Sweden; 3 Department of Clinical Chemistry and Transfusion Medicine, Institute of Biomedicine, Sahlgrenska University Hospital, Gothenburg University, Gothenburg, Sweden

**Keywords:** Gap junction-coupled cells, Astrocytes, Inflammation, Restoration, Ca^2+^ signaling, Na^+^/K^+^-ATPase, TLR4, Glucose, Pharmaceuticals

## Abstract

**Background:**

Pharmaceutical treatment with probable anti-inflammatory substances that attack cells in various ways including receptors, ion channels, or transporter systems may slow down the progression of inflammatory conditions. Astrocytes and microglia are the most prominent target cells for inflammation in the central nervous system. Their responses upon inflammatory stimuli work through the NO/cyclic GMP/protein kinase G systems that can downregulate the ATP-induced Ca^2+^ signaling, as well as G protein activities which alter Na^+^ transporters including Na^+^/K^+^-ATPase pump activity, Toll-like receptor 4 (TLR4), glutamate-induced Ca^2+^ signaling, and release of pro-inflammatory cytokines. The rationale for this project was to investigate a combination of pharmaceutical substances influencing the NO and the G_i_/G_s_ activations of inflammatory reactive cells in order to make the cells return into a more physiological state. The ATP-evoked Ca^2+^ signaling is important maybe due to increased ATP release and subsequent activation of purinergic receptors. A balance between intercellular Ca^2+^ signaling through gap junctions and extracellular signaling mediated by extracellular ATP may be important for physiological function.

**Methods:**

Astrocytes in primary cultures were incubated with lipopolysaccharide in a physiological glucose concentration for 24 h to induce inflammatory reactivity. The probable anti-inflammatory substances sildenafil and 1α,25-Dihydroxyvitamin D3 together with endomorphin-1, naloxone, and levetiracetam, were used in the presence of high glucose concentration in the medium to restore the cells. Glutamate-, 5-HT-, and ATP-evoked intracellular Ca^2+^ release, Na^+^/K^+^-ATPase expression, expression of inflammatory receptors, and release of tumor necrosis factor alpha were measured.

**Results:**

Sildenafil in ultralow concentration together with 1α,25-Dihydroxyvitamin D3 showed most prominent effects on the ATP-evoked intracellular Ca^2+^ release. The μ-opioid agonist endomorphin-1, the μ-opioid antagonist naloxone in ultralow concentration, and the antiepileptic agent levetiracetam downregulated the glutamate-evoked intracellular Ca^2+^ release and TLR4. The combination of the pharmaceutical substances in high glucose concentration downregulated the glutamate- and ATP-evoked Ca^2+^ signaling and the TLR4 expression and upregulated the Na^+^/K^+^-ATPase pump.

**Conclusion:**

Pharmaceutical treatment with the combination of substances that have potential anti-inflammatory effects, which attack different biochemical mechanisms in the cells may exert decisive effects to downregulate neuroinflammation in the nervous system.

## Background

Substantial progress is made in understanding the interplay between chronic low-grade systemic inflammation and diseases such as neurodegenerative, metabolic, or autoimmune diseases and even stroke and traumatic brain injury. Blood-brain barrier (BBB) breakdown is a critical event in inflammation in the central nervous system (CNS), and immune cells are involved in defense mechanisms against pathogens [1, 2]. Microglia and astrocytes are responsible for the innate immune response in the CNS, and their activation constitutes one of the most notable characteristics of neuroinflammation. Inflammatory processes are initiated when the glial cells are activated [3]. The astrocytes are excitable but do not express action potentials. They are equipped with Ca^2+^ signaling systems, which can be inter- and/or extracellular, and transport of small molecules between the cells occurs through gap junctions [4–6].

It is well known that astrocytes upon inflammatory processes are affected with an over-activation in Ca^2+^ signaling, which triggers astrocytes and microglia to become inflammatory reactive [7]. Thereby there will be downregulations of Na^+^ transporters and disruption of the cytoskeleton. Furthermore, the release of pro-inflammatory cytokines increases. There is increased neuronal excitability and increased glutamate release. Astrocyte uptake of excessive extracellular glutamate takes place through glutamate transporters, and ionotropic and metabotropic glutamate receptors are activated [8–10]. Increased inflow of Ca^2+^ occurs through the N-methyl-D-aspartate (NMDA) receptor [11]. The complex Ca^2+^/calmodulin activates nitric oxide synthases, which converts L-arginine to nitric oxide (NO) resulting in accumulation of cyclic GMP and activation of protein kinase G (PKG) [12]. Cyclic GMP is fast hydrolyzed by phosphodiesterases (PDEs), where PDE-5 plays a central role [3]. PDE inhibitors exert a direct anti-inflammatory effect by raising cyclic GMP. There are indications that the NO/cyclic GMP/PKG pathway is the central signaling mechanism and can thereby be a potential tool in diseases where inflammation, including neuroinflammatory disorders, play a central role [3, 13, 14]. The potent and selective PDE-5 inhibitor sildenafil induces cyclic GMP accumulation, which may inhibit inflammation [14]. It has been used in therapy in erectile dysfunction [15] and pulmonary hypertension [16], and it affects neuronal survival [17]. Sildenafil can also normalize endothelial function and has been proposed for pain therapy in humans and animals [18] due to its anti-inflammatory properties. Furthermore, our group has shown that sildenafil works as an anti-inflammatory substance in lipopolysaccharide (LPS)-induced inflammatory reactive astrocytes and we observed that ATP-evoked intracellular Ca^2+^ release was prominently affected [19].

The opioid antagonist naloxone in ultralow concentrations inhibits the G_s_ protein and activates the Na^+^/K^+^-ATPase activity. The opioid agonist endomorphin-1 activates the G_i/o_ protein, and the anti-epileptic agent levetiracetam decreases the release of IL-1β [20, 21]. In inflammatory reactive astrocytes, this pharmaceutical combination was earlier shown to restore the disorganized actin filaments [21]. 1α,25-Dihydroxyvitamin D3 (vitamin D3) acts as an immune regulator to protect against BBB disruption [22], downregulates TLR4, and decreases TNF-α and IL-6 release [23, 24].

We cultivated astrocytes in two different glucose concentrations—physiological or high glucose—and compared the different cultures after stimulation with sildenafil. The hypothesis is that cells grown in physiological glucose concentration increase the inflammatory reactivity better than cells grown in high glucose concentration. High glucose might have anti-inflammatory properties [25].

The first purpose of this study was to evaluate the anti-inflammatory effects of sildenafil (Viagra®) in glial cells. The hypothesis is that the NO/cyclic GMP/protein kinase G systems can downregulate intracellular Ca^2+^ release evoked by ATP and maybe also have effects on inflammatory receptors. We tested the effects on cells cultivated in low and high glucose concentrations, respectively.

The second purpose was to evaluate the effects of the combination of the μ-opioid receptor antagonist, naloxone, at ultralow concentrations, the μ-opioid receptor agonist, endomorphin-1, and levetiracetam, earlier shown by us to affect glutamate-evoked intracellular Ca^2+^ release [21, 26]. This was done in combination with sildenafil in ultralow concentration and vitamin D3, which affect ATP-evoked intracellular Ca^2+^ release. Our hypothesis is that inflammatory processes influence the glutamate- and ATP-induced Ca^2+^ release as well as inflammatory receptors and the Na^+^/K^+^-ATPase pump activity. Ca^2+^ responses to ATP stimulation is changed due to inflammation and mediated by extracellular diffusion of ATP leading to activation of purinergic receptors. Thus, we want to affect these biochemical systems with substances, which have probable anti-inflammatory effects in the nervous system.

## Methods

### Cell model system

Primary cortical astrocytes from Sprague Dawley newborn rats were purchased from 3H Biomedical Science (Uppsala, Sweden) and prepared according to the manufacturer’s instructions with some modifications [8, 21]. The cultures were delivered in 5.5 mM glucose and cultivated in 5.5 mM glucose (normal concentration) the whole period. In some experiments, the cells were cultivated in 25 mM glucose (high concentration) the whole period.

### LPS treatment and pharmaceutical restoration

To evaluate an optimal LPS concentration, a concentration curve including 0, 1, 10, 100, 500, and 1000 ng/ml evaluated by tumor necrosis factor alpha (TNF-α) release was used. The cells were incubated for 24 h.

For anti-inflammatory restoration, the cell cultures were incubated with LPS for 24 h. Restoration proceeds so that the cells are incubated with the pharmaceutical substances together with LPS for further 24 h. Substances are naloxone (10^−12^ M) (Sigma Aldrich, St. Louis, MO, USA), endomorphin-1 (10^−6^ M) (Sigma Aldrich), levetiracetam (10^−4^ M) (Sigma Aldrich), sildenafil citrate salt (1 μM) (Sigma Aldrich), and 1α,25-Dihydroxyvitamin D3 (100 nM) (Sigma Aldrich).

### Cytokine release

TNF-α (BD Biosciences, San Diego, USA) was used according to the manufacturer’s instructions to measure the amount of cytokine released with ELISA. Between every incubation step, several washes were performed. The amount of TNF-α release was normalized to the protein content.

### Immunocytochemistry

The cells were fixed with 4% paraformaldehyde (Bie & Berntsen, Herlev, Denmark) for 10 min and washed twice with phosphate buffer saline (PBS) (Invitrogen, Carlsbad, USA) containing 1% BSA (PBS-BSA). The cells were permeabilized with PBS-BSA containing 0.05% saponine (PBS-BSA-Sap) for 20 min. Thereafter, the cells were incubated for 1 h with a cocktail of rabbit polyclonal antibody against glial fibrillary acidic protein (GFAP) (Dako, Glostrup, Denmark) and a mouse monoclonal antibody against OX42 (Serotec Oxford, UK). Both antibodies were diluted 1:100 in PBS-BSA-Sap. The cells were washed with PBS-BSA-Sap for 3 × 5 min and then incubated with a mixture of FITC-conjugated F(ab´)_2_ fragment donkey anti-mouse IgG and a Dylight 594-conjugated F(ab´)_2_ fragment donkey anti-rabbit IgG secondary antibodies (Jackson ImmunoResearch Europe Ltd., Suffolk, UK), both diluted 1:150. The cells were washed with PBS-BSA-Sap for 3 × 5 min and finally rinsed with PBS. The cover slips were mounted on microscope slides with a fluorescent mounting medium (Dako) and viewed in a Nikon Eclipse 80*i* microscope. Pictures were taken with a Hamamatsu C5810 colour intensified 3CCD camera.

### Cell counting

Regions for cell counting were sampled randomly. In each culture slide, five areas were chosen randomly and three cultures from each type of experiments were done to ensure any differences in the cultures.

### Calcium imaging

With a high-throughput screening system for intracellular Ca^2+^ signaling, Flexstation 3 Microplate Reader (Molecular Devices, San José, USA), the cells were incubated with the Ca^2+^-sensitive probe FLIPR Calcium 6 (Molecular Devices) and stimulated immediately before the experiment started with different neurotransmitters—5-HT (10^− 5^ M), glutamate (10^− 3^ M), or ATP (10^− 4^ M) all from Sigma Aldrich (Saint Louis, USA). The intracellular Ca^2+^ release was determined as the total areas under the curve (AUC), which reflects the amount of Ca^2+^ released [27]. The amplitude (peak) was expressed as the maximum increase.

### SDS-PAGE and Western blotting

The cells were rinsed twice in phosphate buffered saline (PBS) and immediately lysed for 20 min on ice in cold radio-immunoprecipitation assay (RIPA) lysis buffer containing 150 mM NaCl, 1% IGEPAL® CA-630, 0.5% sodium deoxycholate, 0.1% SDS, 50 mM Tris, (pH 8.0), supplemented with a protease inhibitor cocktail containing 104 mM AEBSF, 80 μM aprotinin, 4 mM bestatin, 1.4 mM E-64, 2 mM leupeptin, and 1.5 mM pepstatin A. The procedure was performed according to Persson et al. [28]. Separate aliquots were collected to determine the protein concentration. All of the samples were analyzed for the total protein content, and 20 μg of the total protein from each sample was loaded into each lane of the gel. β-Actin was used as a control for equal loading.

SDS-PAGE was conducted using the Novex pre-cast gel system (Invitrogen) according to the manufacturer’s recommendations using 4–12% Bis-Tris gels (Invitrogen) at 200 V for 50 min. The separated proteins were transferred at 30 V for 60 min to a nitrocellulose membrane (Invitrogen) using NuPAGE transfer buffer (Invitrogen) supplemented with methanol and NuPAGE antioxidant. The membranes were rinsed twice with distilled water, and the proteins were visualized with Ponceau S solution (Sigma Aldrich). The proteins were blocked with 0.5% fat-free skim milk (Semper AB, Götene, Sweden) in Tris-buffered saline (TBST; 50 mM Tris-HCl, 150 mM NaCl, and 0.05% Tween) for 60 min at room temperature. The membranes were probed with anti-TLR4 (rabbit polyclonal, 1:500) (Santa Cruz Biotech Inc., Dallas, TX, USA), anti-NK-1 (rabbit polyclonal, 1:1000) (LifeSpan BioSciences Inc., Seattle, USA), anti-PAR-2 (rabbit polyclonal, Santa Cruz Biotech Inc), or a mouse monoclonal primary antibody against Na^+^/K^+^-ATPase (α-subunit) (Sigma Aldrich) diluted 1:250, washed four time for 2 min with TBST, followed with the secondary horseradish peroxidase (HRP)-conjugated antibodies, donkey anti-mouse, or anti-rabbit F(ab’)_2_ fragment (Jackson ImmunoResearch) diluted 1:10000, also washed several times in TBST. All of the primary and secondary antibodies were diluted in 0.5% fat-free skim milk in TBST. The antibody-bound protein was detected with an enhanced chemiluminescence kit (PerkinElmer Inc., Waltham, MA, USA) and visualized using Fuji Film LAS-3000 (Tokyo, Japan).

### Protein determination

The protein determination assay was performed in accordance with the manufacturer’s instructions using a detergent-compatible (DC) Protein Assay (Bio-Rad, Hercules, CA, USA) based on the method used by Lowry and co-workers [29] with minor modifications. The standard (0–4 mg/ml BSA) and samples were mixed with the reagents, incubated for 15 min at room temperature, read at 750 nm with a Versa-max microplate reader, and analyzed using SoftMax Pro 4.8 from Molecular Devices (Sunnyvale, CA, USA).

### Statistical analysis

Differences across the different treatments were identified using a one-way ANOVA followed by Dunnett’s multiple comparisons test. The error bars represent the standard error of the mean (SEM).

## Results

### Astroglial cultures

**Fig. 1:**
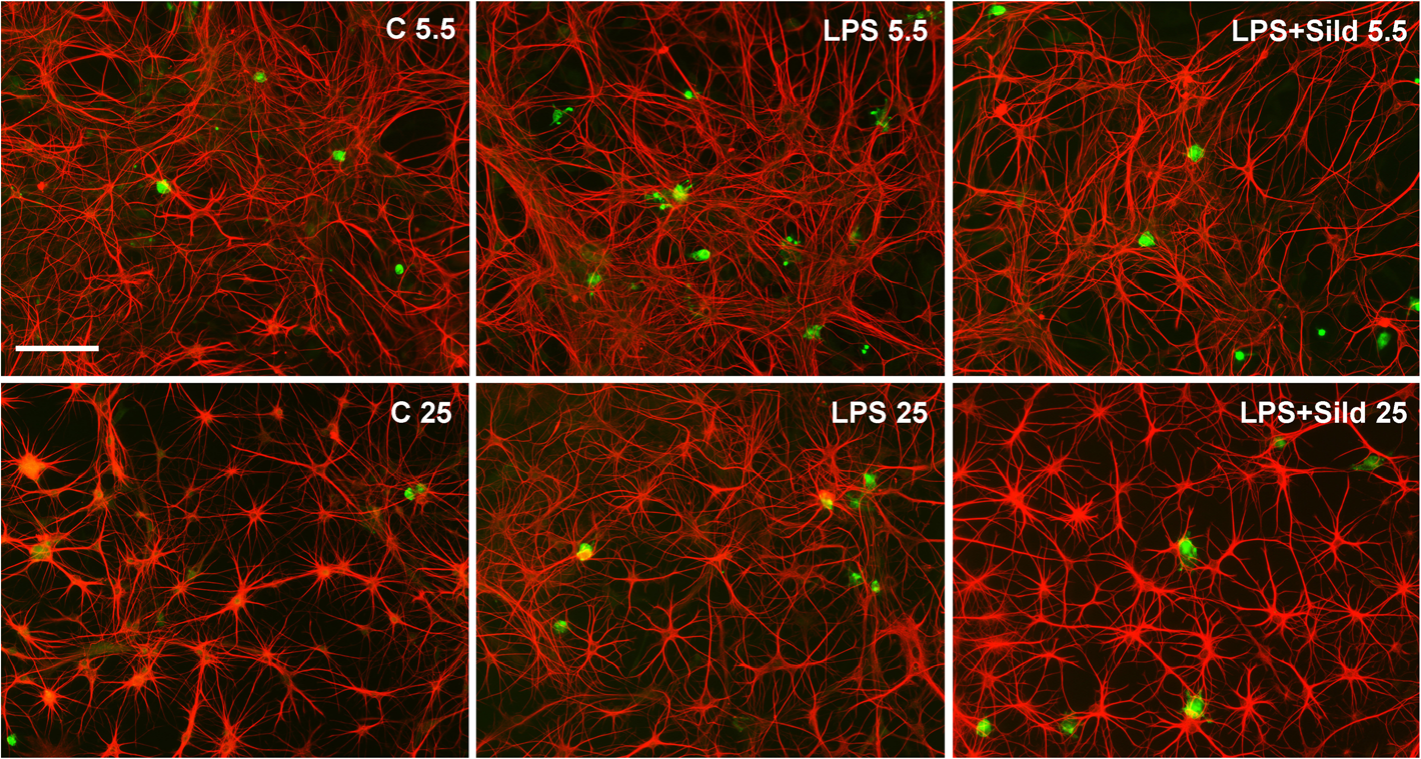
Culture stained for OX42, a marker for microglial cells (green), and GFAP, a marker for astrocytes (red). Some microglia, less than 5%, were found in the control cultures ©, 5.5 mM glucose. The amount of microglia increased to 5–10% after treatment with LPS for 24 h in 5.5 mM glucose. The amount of microglia decreased after treatment with LPS for 24 h followed by incubation with LPS and sildenafil (Sild) for another 24 h in 5.5 mM glucose to less than 5%. Cultures cultivated in 25 mM glucose were less affected, 1–2%. Scale bar = 50 μm. Representative images are presented

The astroglial cultures contain some few percents of microglial cells, less than 5% when bought from the supplier. It has turned out through our experiments the necessity to have a critical number of microglia in the astroglial cultures to obtain reactive inflammatory astrocytes after incubation with LPS. The amount of microglia increased to 5–10% when the cultures were incubated with LPS (10 ng/ml) for 24 h. When the cultures were incubated first with LPS for 24 h followed by incubation with LPS and sildenafil (1 μM) [19] for another 24 h, the number of microglial cells decreased, less than 5% (Fig. 1).

### LPS treatment

**Fig. 2:**
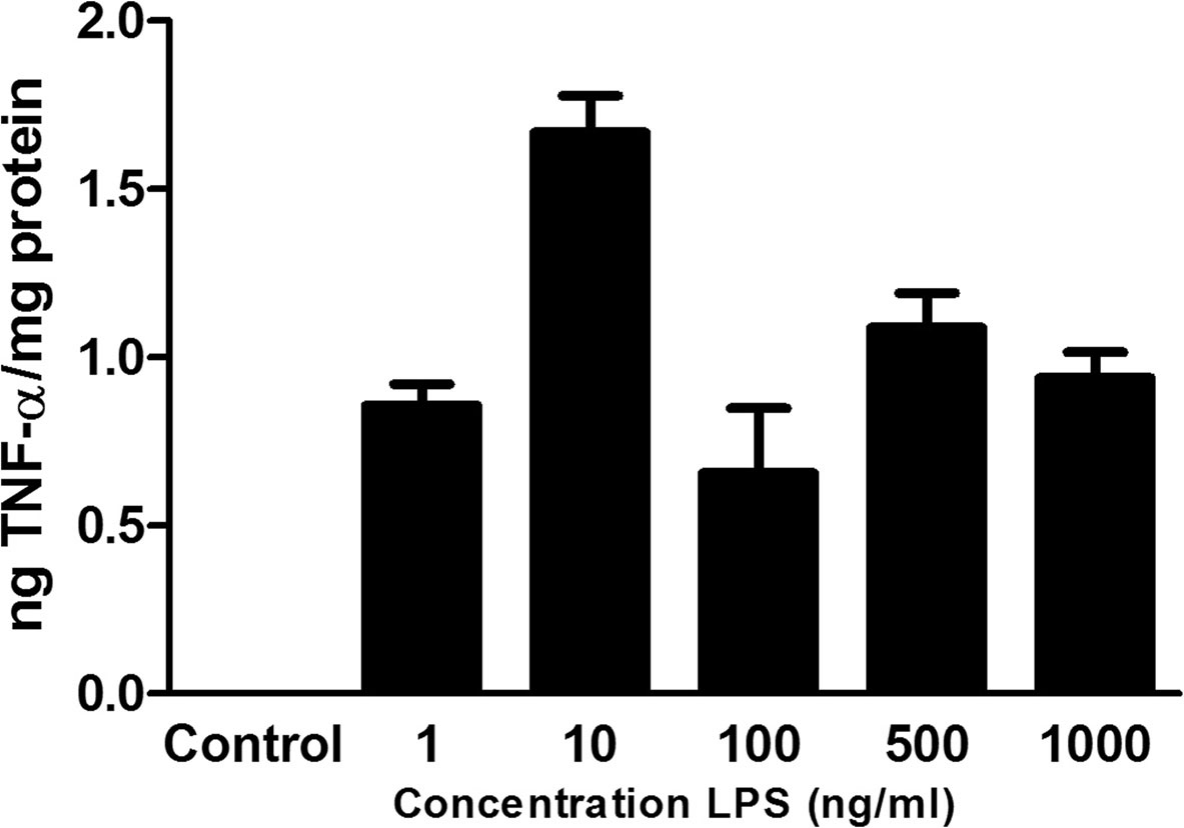
LPS concentration curve verified by TNF-α release. *n* = 6

The LPS concentration was evaluated with a concentration curve. 10 ng/ml LPS turned out to be the optimal concentration verified by TNF-α release (Fig. 2). In all further experiments, 10 ng/ml LPS were used.

### Evaluation of physiological and high glucose concentration

**Fig. 3:**
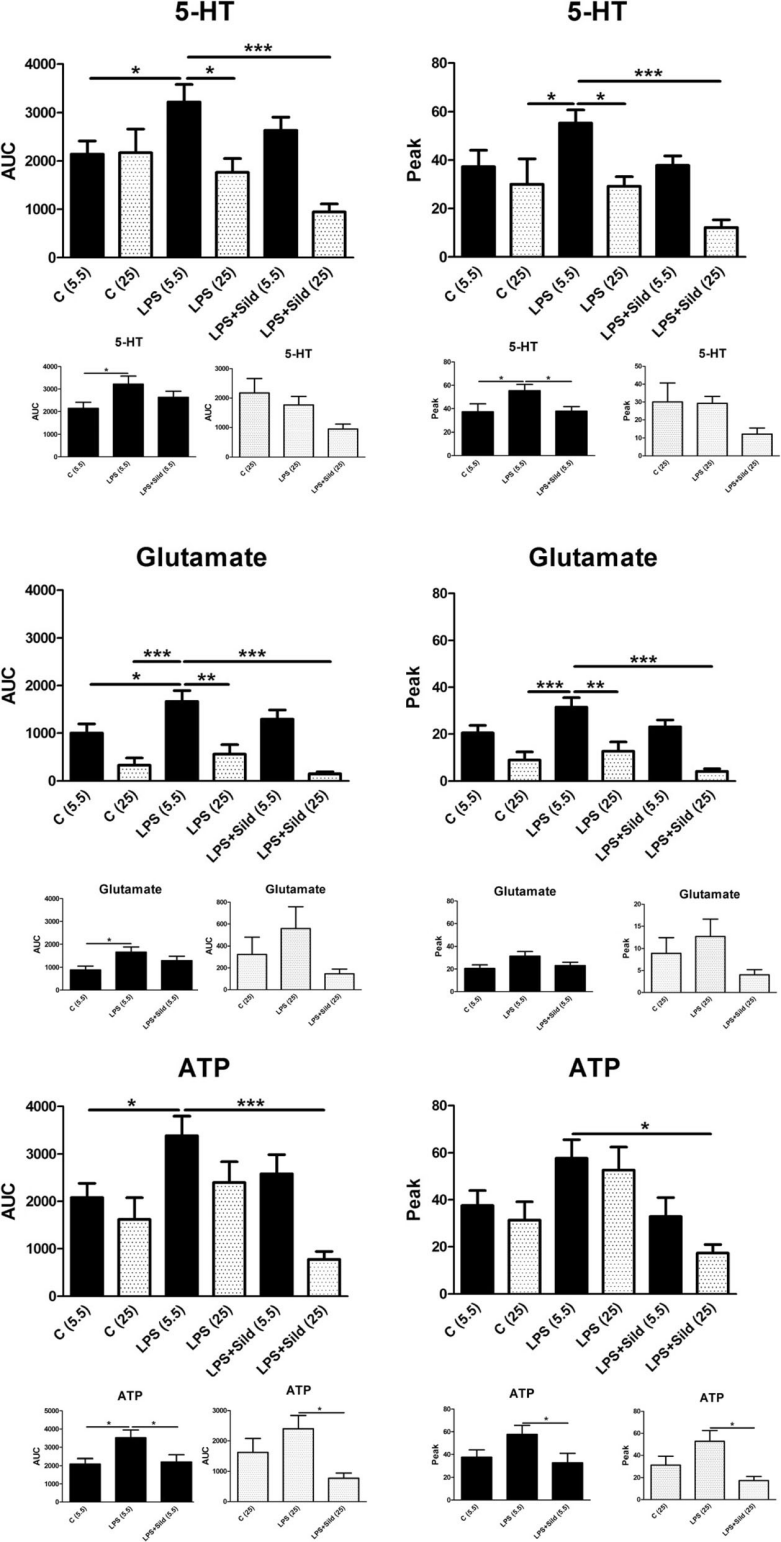
Astrocytes were stimulated, in a fluorescence-based assay for detecting changes in intracellular Ca^2+^ over time, with the following: 5-HT (10^−5^ M), glutamate (10^−3^ M), or ATP (10^−4^ M). The cells were cultivated in 5.5 mM or 25 mM glucose the whole cultivation period. Ca^2+^ responses when incubated with LPS (10 ng/ml) for 24 h, or when incubated with LPS for 24 h followed by LPS and sildenafil (Sild) (1 μM); unstimulated cells were used as controls ©. The area under the Ca^2+^ peak (AUC) was calculated for each Ca^2+^ transient, and the amplitude (peak) was expressed as the maximum increase. The cells were obtained from three experiments with quadruple wells in each. The level of significance was calculated against LPS (5.5) and analyzed using one-way ANOVA followed by Dunnett’s multiple comparisons test. *P < 0.05, **P < 0.01, ***P < 0.001. Separate small histograms show cells cultivated in 5.5 mM glucose and 25 mM glucose, respectively. The level of significance was calculated against LPS (5.5) or against LPS (25). *P < 0.05

**Fig. 4:**
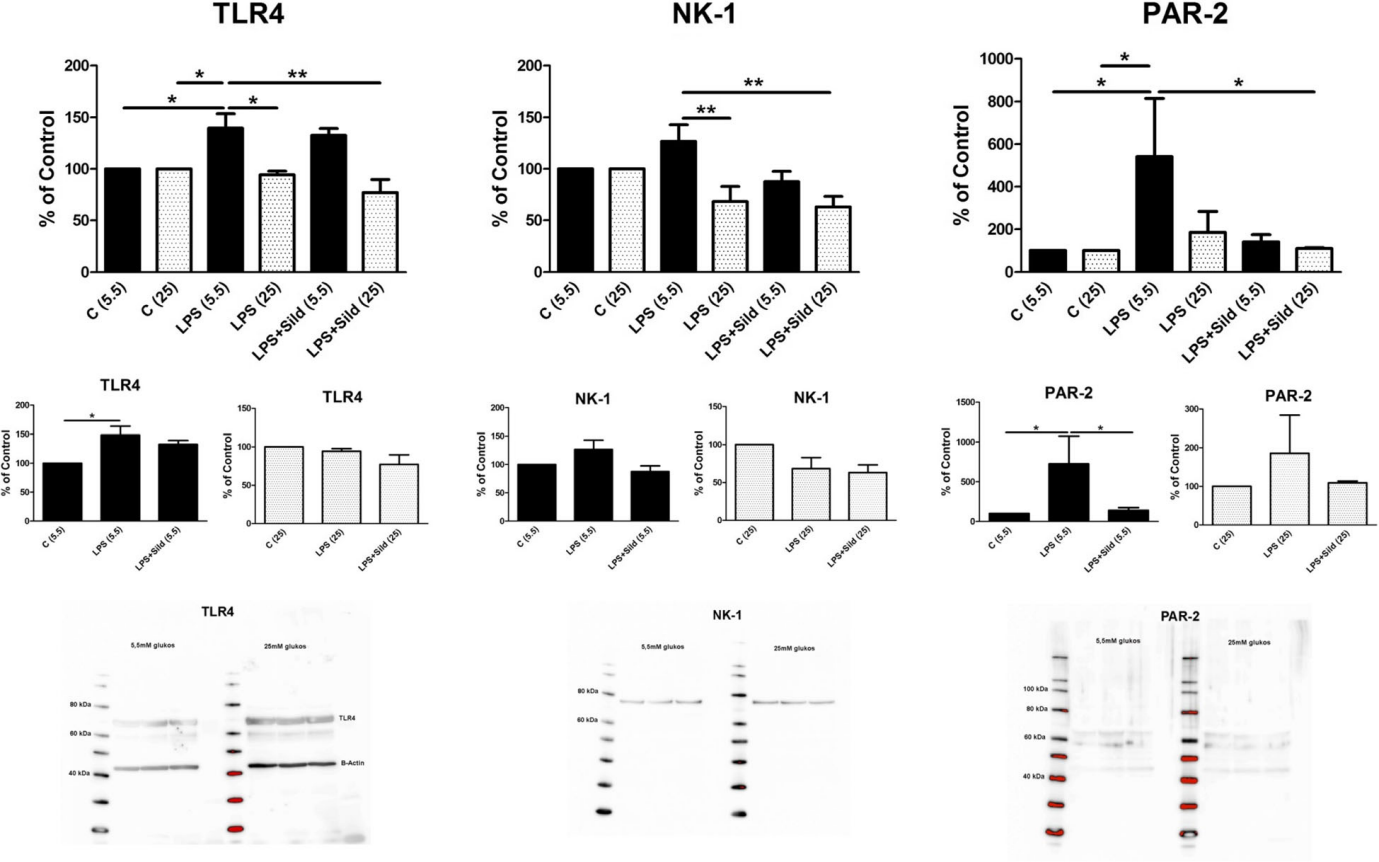
Expression levels of the receptors TLR4, NK-1, and PAR-2 were studied using Western blot analysis. Astrocytes were cultivated in 5.5 mM or 25 mM glucose the whole cultivation period. The astrocytes were incubated with LPS (10 ng/ml) for 24 h or incubated with LPS for 24 h followed by LPS and sildenafil (Sild) (1 μM), or combination of all substances for another 24 h; unstimulated cells were used as controls ©. Statistical analysis: the level of significance was calculated against LPS (5.5) and analyzed using one-way ANOVA followed by Dunnett’s multiple comparisons test. *P < 0.05, **P < 0.01. *n* = 6. Separate small histograms show cells cultivated in 5.5 mM glucose and 25 mM glucose, respectively. The level of significance was calculated against LPS (5.5) or against LPS (25). *P < 0.05. Representative images of Western blot membranes are presented

Astrocytes in culture, cultivated in 5.5 mM glucose physiological concentration, achieve inflammatory reactivity when incubated with LPS for 24 h. Astrocytes cultivated in high glucose concentration, 25 mM, were very difficult or impossible to achieve inflammatory reactivity. This is visualized with the intracellular evoked Ca^2+^ release (Fig. 3) and expression of several inflammatory receptors such as the TLR4, the substance P receptor (NK-1), and the tryptase receptor (PAR-2) (Fig. 4).

Referring to intracellular evoked Ca^2+^ release, no differences were obtained in the control cultures cultivated in physiological or high glucose concentration during the whole cultivation period (Fig. 3). When incubated with LPS for 24 h, only those cultures cultivated in 5.5 mM glucose evoked increased Ca^2+^ release after the cells were stimulated with 5-HT, glutamate, or ATP (Fig. 3).

Concerning expression of the inflammatory receptors TLR4, NK-1, and PAR-2 (Fig. 4), the cells cultivated in physiological glucose concentration increased their expression of the inflammatory receptors when incubated with LPS for 24 h. Cultivation in high glucose did not change the expression of either inflammatory receptor (Fig. 4).

### Restoration of inflammatory reactive astrocytes cultivated in high glucose concentration

An extremely low concentration of sildenafil, 1 μM, was found to be optimal for ATP-evoked intracellular Ca^2+^ release [19]. For anti-inflammatory restoration back to a normal physiological level, the cells were first incubated with LPS for 24 h followed by LPS + sildenafil for further 24 h. The cells were cultivated in physiological or high glucose concentrations from start.

Cells cultivated in 5.5 mM glucose and stimulated with LPS reached inflammatory reactivity. However, cells cultivated in 25 mM glucose attenuated the 5-HT-, glutamate- and ATP-evoked Ca^2+^ release compared to LPS incubated inflammatory cells cultivated in 5.5 mM glucose (Fig. 3).

The expression of TLR4, NK-1, or PAR-2 receptors decreased when the cells were cultivated in 25 mM glucose, stimulated with LPS and sildenafil compared to cells cultivated in 5.5 mM glucose and stimulated with LPS (Fig. 4). The results show that sildenafil decreased inflammatory markers.

**Fig. 5:**
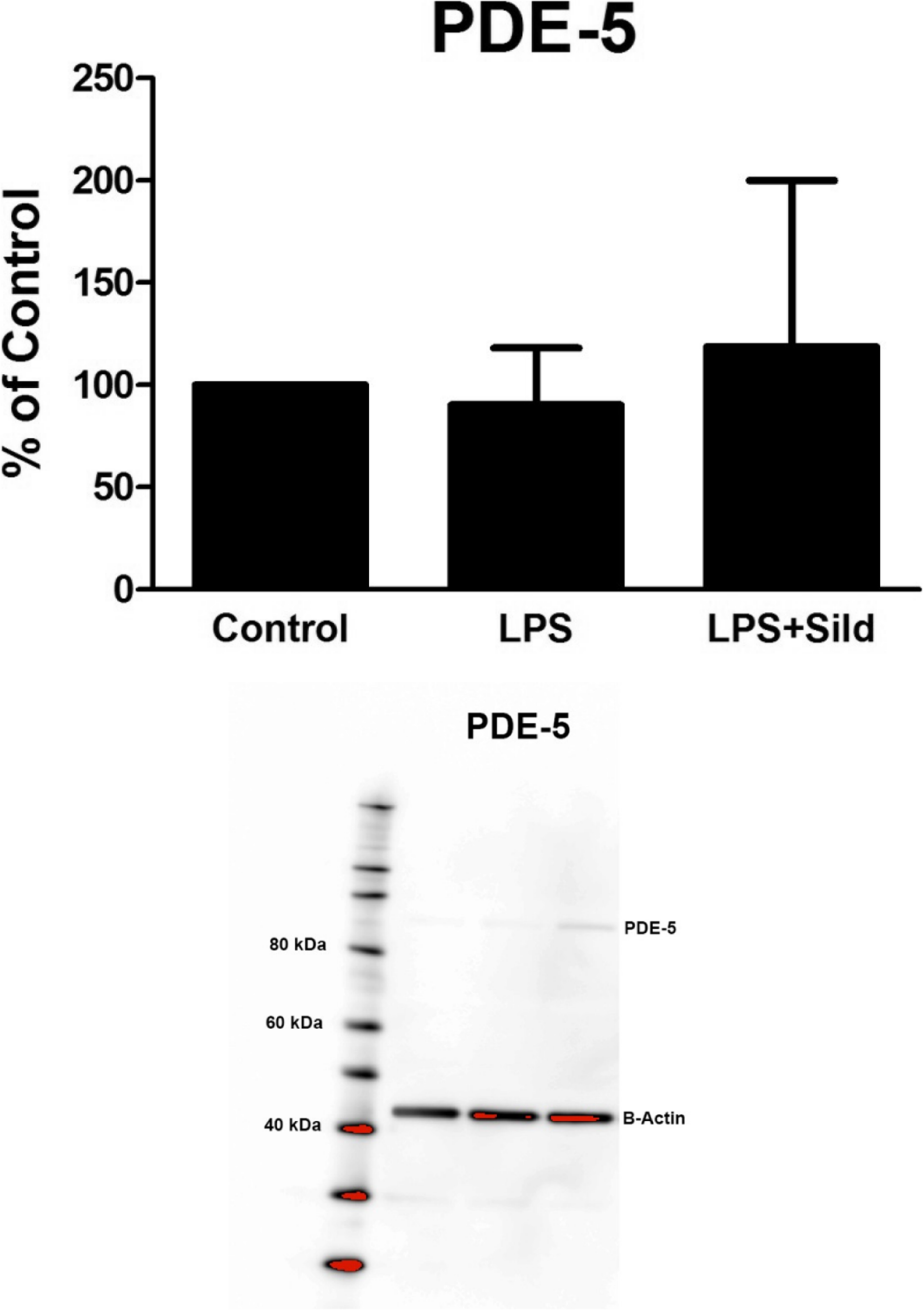
The expression levels of PDE-5 were studied using Western blot analysis. Astrocytes were cultivated in 5.5 mM glucose the whole cultivation period. The astrocytes were incubated with LPS (10 ng/ml) for 24 h or incubated with LPS for 24 h followed by LPS and sildenafil (Sild) (1 μM) for another 24 h. *n* = 3. Representative image of Western blot membrane is presented

As sildenafil is a potent and selective PDE-5 inhibitor, we show that our astrocytes also express PDE-5, which were not changed in expression after the cells were incubated with LPS (Fig. 5).

### Restoration of inflammatory reactive astrocytes cultivated in physiological glucose concentration

Restoration of inflammation-reactive astrocytes with a combination of three substances—an opioid agonist, an opioid antagonist, and an anti-epileptic substance—has earlier been tested. These results showed that only the glutamate-induced Ca^2+^ signaling was influenced [21, 26]. We hypothesize that even the ATP-evoked Ca^2+^ signaling is important maybe due to increased ATP release [7] and subsequent activation of purinergic receptors. A balance between intercellular Ca^2+^ signaling through gap junctions and extracellular signaling mediated by extracellular ATP is preferred.

Astrocytes in the following experiments were all cultivated in 5.5 mM glucose from the start. To become inflammatory reactive, the cells were incubated with LPS for 24 h. For restoration, the cells continued with LPS + addition of high glucose together with pharmaceuticals in combination for additional 24 h: (1) the μ-opioid antagonist naloxone in ultralow concentration, the μ-opioid agonist endomorphin-1, and the anti-epileptic agent levetiracetam, (2) ultralow concentration of sildenafil and vitamin D3, (3) combination (1) and (2).

**Fig. 6:**
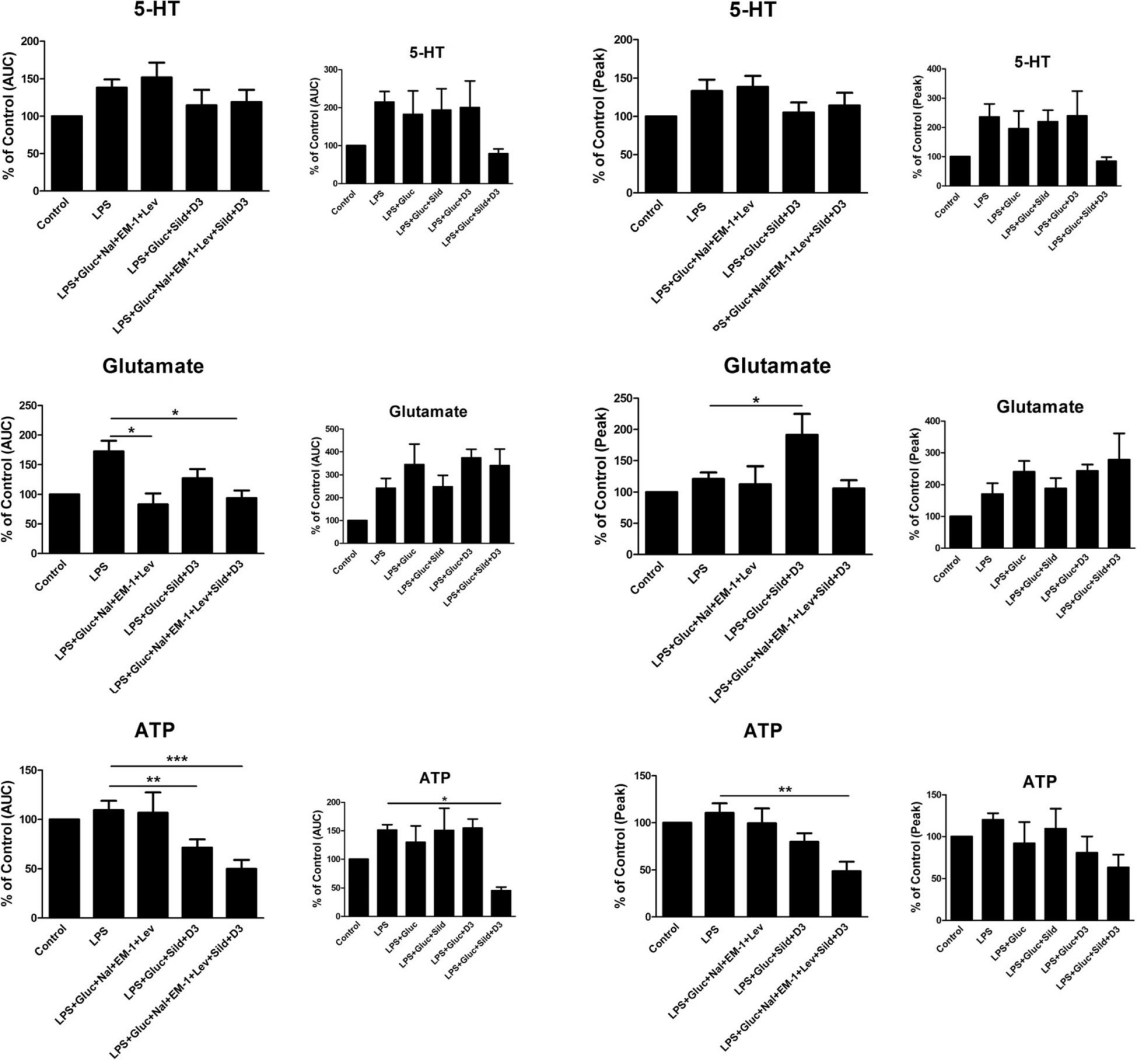
Astrocytes were stimulated, in a fluorescence-based assay for detecting changes in intracellular Ca^2+^ over time, with the following: 5-HT(10^−5^ M), glutamate (10^−3^ M), or ATP (10^−4^ M). AUC and peak values of Ca^2+^ transients are shown. The cells were cultivated in 5.5 mM glucose the whole cultivation period. Ca^2+^ responses were measured after the cells were incubated with LPS (10 ng/ml) for 24 h, when incubated with LPS for 24 h followed by LPS, 25 mM glucose, a combination of naloxone (Nal) (10^−12^ M), endomorphin-1 (EM-1) (10^−6^ M), and levetiracetam (Lev) (10^−4^ M), or a combination of sildenafil (Sild) (1 μM) and vitamin D3 (D3) (100 nM) or combination of all substances for another 24 h. Unstimulated cells were used as controls. The area under the Ca^2+^ peak (AUC) was calculated for each Ca^2+^ transient, and the amplitude (peak) was expressed as the maximum increase. The cells were obtained from four experiments with quadruple wells in each. The level of significance was calculated against LPS (5.5) and analyzed using one-way ANOVA followed by Dunnett’s multiple comparisons test. *P < 0.05, **P < 0.01, ***P < 0.001. Additional data, small histograms, for LPS + glucose, LPS + glucose + sildenafil, LPS + glucose + vitamin D3, and LPS + glucose + sildenafil + vitamin D3. * *P* < 0.05

The glutamate-evoked intracellular Ca^2+^ release was restored back to a normal Ca^2+^ signaling with the combination of the μ-opioid antagonist naloxone in ultralow concentration, the μ-opioid agonist endomorphin-1, and the anti-epileptic agent levetiracetam (Fig. 6). The ATP evoked intracellular Ca^2+^ release was restored back to a normal Ca^2+^ signaling with the combination of sildenafil and vitamin D3 (Fig. 6).

The combination of high glucose concentration together with ultralow concentration naloxone, endomorphin-1, levetiracetam, ultralow concentration sildenafil, and vitamin D3 restored the inflammatory induced astrocytes back to a physiological Ca^2+^ signaling regarding both the glutamate- and ATP-evoked Ca^2+^ signaling. The 5-HT-evoked Ca^2+^ signaling was not affected (Fig. 6). Additional control data for LPS + glucose, LPS + glucose + sildenafil, LPS + glucose + vitamin D3, and LPS + glucose + sildenafil + vitamin D3 for clarification to Fig. 6 are shown (Fig. 6).

**Fig. 7:**
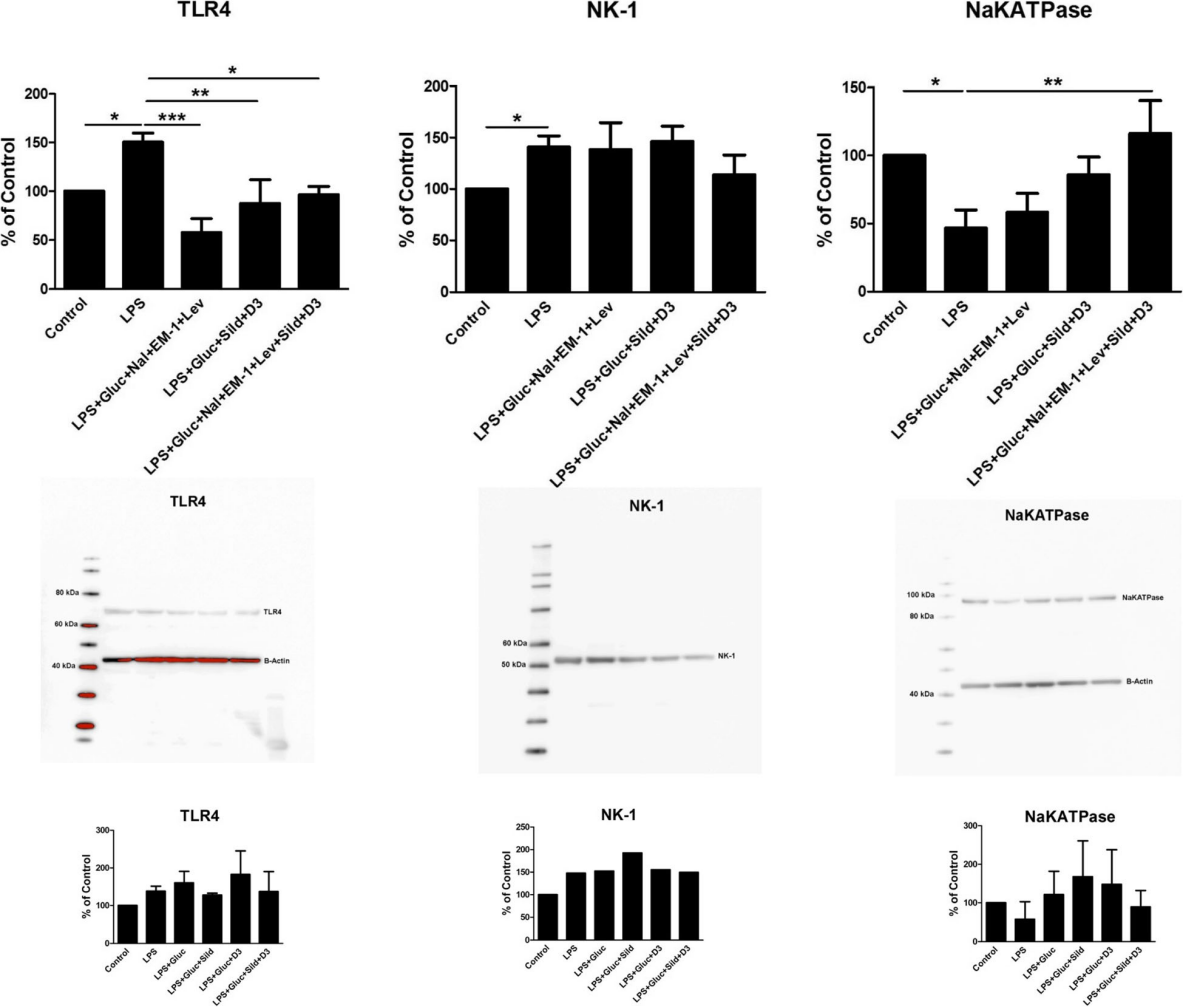
Expression levels of TLR4, NK-1, and Na^+^/K^+^-ATPase were studied using Western blot analysis. Astrocytes were cultivated in 5.5 mM glucose the whole cultivation period. The cells were incubated with LPS (10 ng/ml) for 24 h, when incubated with LPS for 24 h followed by LPS, 25 mM glucose, a combination of naloxone (Nal) (10^− 12^ M), endomorphin-1 (EM-1) (10^− 6^ M), and levetiracetam (Lev) (10^− 4^ M), or a combination of sildenafil (Sild) (1 μM) and vitamin D3 (D3) (100 nM), or combination of all substances for another 24 h. Unstimulated cells were used as controls. The level of significance was calculated against LPS (5.5) and analyzed using one-way ANOVA followed by Dunnett’s multiple comparisons test. *P < 0.05, **P < 0.01, ***P < 0.001. *n* = 6. Representative images of Western blot membranes are presented. Additional data, small histograms below, show LPS + glucose, LPS + glucose + sildenafil, LPS + glucose + vitamin D3, and LPS + glucose + sildenafil + vitamin D3

Expression of the receptors TLR4 and NK-1 showed that TLR4 was attenuated by combination 1, 2, and 1 + 2. No effect was seen with the NK-1 receptor (Fig. 7).

Expression of Na^+^/K^+^-ATPase increased after stimulation with the combination 1 + 2 (Fig. 7). Additional control data for LPS + glucose, LPS + glucose + sildenafil, LPS + glucose + vitamin D3, and LPS + glucose + sildenafil + vitamin D3 for clarification to Fig. 7 are shown (Fig. 7).

## Discussion

Gap junction coupled cellular networks can be target cells leading to spread of inflammation and changes in biochemical cellular parameters. Astrocytes in the CNS are the most well-studied network coupled cells in the body [30], which play a pivotal role in chronic neuroinflammation [31, 32]. Aims are to restore inflammatory influenced coupled cell networks back to a physiological level.

Different pharmaceutical combinations were shown to exert powerful anti-inflammatory effects on the astrocytes in culture. Sildenafil in ultralow concentration showed prominent effects on the ATP-evoked intracellular Ca^2+^ release in accordance with our previous results [19]. The combination of sildenafil and vitamin D3 showed even better effects. The main effect of the combination of the μ-opioid receptor antagonist naloxone in ultralow concentrations, the μ-opioid receptor agonist, endomorphin-1, and levetiracetam was a decrease in the glutamate-evoked intracellular Ca^2+^ release [21] and a reinforcement of the Na^+^ transporters including the Na^+^/K^+^ ATPase and downregulation of TLR4. Clinical tests in patients with naloxone in ultralow concentrations in combination with morphine have shown promising results [33] as anti-inflammatory pharmaceuticals.

Astrocytes in culture were cultivated during the whole period in either physiological glucose concentration, 5.5 mM, or in high glucose concentration, 25 mM. Commercial cell batches are delivered in high glucose, probably to achieve viable and more stable cells. However, we experienced difficulties to achieve inflammatory reactivity with LPS in cells cultivated in high glucose. Therefore, we ordered cells cultivated in 5.5 mM glucose. Inflammatory reactivity was easier to induce in physiological glucose, which we verified concerning 5-HT-, glutamate- and ATP-evoked Ca^2+^ signaling, and expression with the inflammatory receptors TLR4, NK-1, and PAR-2. The results suggest that cultivation in high glucose at least partly protected the cells from inflammatory reactivity.

The number of microglial cells in the cultures seems important for the possibility to induce inflammatory reactive astrocytes, and a minimum number of microglia has been shown to be approximately 5%. Due to this, it can be discussed whether or not the biochemical responses obtained in this paper is dependent on microglial or astroglial activation, or both. It is plausible that microglial activation influences upon the astroglial biochemical machinery, but the amount of microglia in the cultures were too small to be responsible for the overall responses and results obtained. In addition, it should be noted that the Ca^2+^ experiments were performed on single astroglial cells. This matter has been evaluated and discussed earlier by us [8].

One question is whether or not there is a decreased glucose uptake in inflammatory reactive astrocytes depending on dysfunctioning glucose protein transporters, which can in turn lead to the development of cell starvation. If the glucose uptake does not work in a proper way in astrocytes, glucose degradation, glycolysis, an important function that supports signaling mechanisms in the brain, is reduced [34]. Glutamate induces glucose transporter (GLUT1) activity and thereby uptake rates in astrocytes [35]. For this astrocytic metabolism, which consumes ATP, the Na^+^/K^+^-ATPase activity is increased and necessary for intercellular Ca^2+^ waves [36] as well as for intercellular Na^+^ waves [37]. Glucose transport in systemic inflammation and in autoimmune diseases is changed. It is observed that organs other than the brain enhance its glucose uptake, but brain cells react in a different way. It is discussed whether reduced glucose uptake is a result of lower blood flow or insufficient glucose uptake due to subnormal glucose transport [38]. The knowledge on glucose transport and the role of glucose transporters is incomplete and needs further investigations.

If we take the clinical problem into consideration, patients suffering in neuroinflammation may have glucose transporters that do not work in a proper way. A challenge to the problem is to test combinations of pharmaceutical substances that can restore inflammatory cellular parameters. The glial cultures are cultivated in physiological glucose concentration to achieve inflammatory reactivity. In the restoration process, high glucose is added to activate the glucose transporter systems. The LPS-induced astrocytes were stimulated with different pharmaceutical combinations together with high glucose [25].

Sildenafil in ultralow concentrations decreased the ATP-evoked Ca^2+^ signaling [19]. It turned out that the glial cells grown in normal glucose were restored with pharmaceuticals that have anti-inflammatory properties in combination with high glucose.

ATP is involved in many cellular signaling systems and increased ATP release from excitable astrocytes leads to increased extracellular Ca^2+^ signaling [39, 40]. An enhanced extracellular ATP concentration as a result of astrocyte activation is seen in inflammatory diseases [41]. We also received increased ATP-evoked Ca^2+^ signaling in inflammatory induced astrocytes. The ATP-evoked Ca^2+^ signaling decreased to control level with an ultralow concentration of sildenafil in combination with vitamin D3. The potent and selective PDE-5 inhibitor sildenafil [14] has shown to have anti-inflammatory and neuroprotective effects maybe through modulation of AMP-activated protein kinase (AMPK) and NFқB signaling [42]. AMPK also promotes glucose uptake [43]. Furthermore, sildenafil reduces the expression of the pro-inflammatory cytokines IL-1β and TNF-α [44]. It is suggested that the cGMP pathway may regulate responses after inflammatory induction including the cellular cytoskeleton [45].

Vitamin D3, a neuroprotective hormone which activates its receptor vitamin D receptor (VDR), protects against BBB disruption in endothelial cells in microvessels [22]. VDR is expressed in all tissues including astrocytes [46]. Vitamin D3 acts as an immune regulator and a stimulator of neurotrophic factors and neurotransmitter expression [23]. Furthermore, vitamin D3 downregulates TLR4 and decreases TNF-α and IL-6 release [23, 24]. As vitamin D3 attenuates inflammatory responses, we included it in our pharmaceuticals for the restoration of inflammatory reactive astrocytes.

It is well known that μ-opioid receptor agonists stimulate the G_i/o_ protein of the μ-opioid receptor. In the chronic administration of morphine or increased endogenous production of endomorphin, a switch in G protein coupling from G_i/o_ to G_s_ proteins occurs. It is found important to stimulate G_i/o_ proteins and decrease G_s_ protein activities [21]. Naloxone in ultralow concentration inhibits the G_s_ protein. Thereby, a switch of the μ-opioid receptor coupling back to the G_i/o_ proteins occurs. This is discussed in [21]. The anti-epileptic drug levetiracetam decreases interleukin-1β release and has anti-inflammatory properties [20, 21]. The combination, the μ-opioid antagonist naloxone in ultralow concentration, the μ-opioid agonist endomorphin-1, and the anti-epileptic agent levetiracetam, had only effects on the glutamate-evoked intracellular Ca^2+^ release. The 5-HT- and ATP-evoked intracellular Ca^2+^ release does not seem to be affected.

The results show that high glucose together with the ultralow concentration of sildenafil and vitamin D3 attenuated the intracellular Ca^2+^ release evoked by ATP. Also, the inflammatory receptors, TLR4, NK-1, and PAR-2, were decreased in protein expressions. The combination in high glucose together with the μ-opioid antagonist naloxone in ultralow concentration, the μ-opioid agonist endomorphin-1, the anti-epileptic agent levetiracetam, ultralow concentration sildenafil, and vitamin D3 restored both the glutamate- and ATP-evoked intracellular Ca^2+^ release back to a physiological homeostasis. Furthermore, the Na^+^ transporters including the Na^+^/K^+^-ATPase pump were increased and the release of pro-inflammatory cytokines, glutamate release, and TLR4 expression were reduced.

Taken together, our results show that by combining different pharmaceuticals with possible anti-inflammatory actions, important biochemical processes may be influenced upon.

## Conclusions

We show that cultivating astrocytes in low glucose concentration induce inflammatory reactivity after incubation with LPS. Sildenafil in ultralow concentration in combination with vitamin D3 had prominent effects on the ATP-evoked intracellular Ca^2+^ release. The μ-opioid agonist endomorphin-1, the μ-opioid antagonist naloxone in ultralow concentration, and the antiepileptic agent levetiracetam downregulated the glutamate-evoked intracellular Ca^2+^ release and TLR4. The combination of the pharmaceuticals in high glucose downregulated the ATP- and glutamate-evoked Ca^2+^ signaling and the expression of TLR4. The expression of the Na^+^/K^+^-ATPase pump was upregulated.

Our unique results suggest that a combination of pharmaceuticals is necessary to restore vital cellular functions in astrocytes and thus inflammatory processes could be inhibited or restored. Our purpose is now to continue with pre-clinical and clinical trials in vivo.

## Abbreviations

AMPK: AMP-activated protein kinase
AUC: Areas under the curve
BBB: Blood-brain barrier
CNS: Central nervous system
GFAP: Glial fibrillary acidic protein
GLUT1: Glucose transporter
HRP: Horseradish peroxidase
IL-1β: Interleukin -1beta
LPS: Lipopolysaccharide
LTP: Learning-related long-term potentiation
NK-1: Substance P receptor;
NMDA: N-methyl-D-aspartate
NO: Nitric oxide
PAR-2: Tryptase receptor
PBS: Phosphate buffer saline
PDE: Phosphodiesterases
PKG: Protein kinase G
RIPA: Cold radio-immunoprecipitation assay
TBST: Tris-buffered saline
TLR4: Toll-like receptor 4
TNF-α: Tumor necrosis factor alpha
VDR: Vitamin D receptor
